# Characterization of Type 1 Angiotensin II Receptor Activation Induced Dual-Specificity MAPK Phosphatase Gene Expression Changes in Rat Vascular Smooth Muscle Cells

**DOI:** 10.3390/cells10123538

**Published:** 2021-12-15

**Authors:** Janka Borbála Gém, Kinga Bernadett Kovács, Laura Szalai, Gyöngyi Szakadáti, Edit Porkoláb, Bence Szalai, Gábor Turu, András Dávid Tóth, Mária Szekeres, László Hunyady, András Balla

**Affiliations:** 1 Department of Physiology, Faculty of Medicine, Semmelweis University, 1085 Budapest, Hungary; gem.janka@phd.semmelweis.hu (J.B.G.); kovacs.kinga@phd.semmelweis.hu (K.B.K.); laura.szalai42@gmail.com (L.S.); szakadati.gyongyi@gmail.com (G.S.); porkolabeda@gmail.com (E.P.); ben.szalai@gmail.com (B.S.); turu.gabor@med.semmelweis-univ.hu (G.T.); toth.andras1@med.semmelweis-univ.hu (A.D.T.); szekeres.maria@med.semmelweis-univ.hu (M.S.); 2 MTA-SE Laboratory of Molecular Physiology, Hungarian Academy of Sciences and Semmelweis University, 1085 Budapest, Hungary; 3 Department of Internal Medicine and Hematology, Semmelweis University, 1085 Budapest, Hungary; 4 Department of Morphology and Physiology, Faculty of Health Sciences, Semmelweis University, 1085 Budapest, Hungary

**Keywords:** angiotensin II (AngII), dual-specificity MAPK phosphatase (DUSP), epidermal growth factor receptor (EGFR), G protein-coupled receptor (GPCR), mitogen-activated protein kinase (MAPK), type 1 angiotensin receptor (AT_1_-R), vascular smooth muscle cell (VSMC)

## Abstract

Activation of the type I angiotensin receptor (AT1-R) in vascular smooth muscle cells (VSMCs) plays a crucial role in the regulation of blood pressure; however, it is also responsible for the development of pathological conditions such as vascular remodeling, hypertension and atherosclerosis. Stimulation of the VSMC by angiotensin II (AngII) promotes a broad variety of biological effects, including gene expression changes. In this paper, we have taken an integrated approach in which an analysis of AngII-induced gene expression changes has been combined with the use of small-molecule inhibitors and lentiviral-based gene silencing, to characterize the mechanism of signal transduction in response to AngII stimulation in primary rat VSMCs. We carried out Affymetrix GeneChip experiments to analyze the effects of AngII stimulation on gene expression; several genes, including *DUSP5*, *DUSP6*, and *DUSP10*, were identified as upregulated genes in response to stimulation. Since various dual-specificity MAPK phosphatase (DUSP) enzymes are important in the regulation of mitogen-activated protein kinase (MAPK) signaling pathways, these genes have been selected for further analysis. We investigated the kinetics of gene-expression changes and the possible signal transduction processes that lead to altered expression changes after AngII stimulation. Our data shows that the upregulated genes can be stimulated through multiple and synergistic signal transduction pathways. We have also found in our gene-silencing experiments that epidermal growth factor receptor (EGFR) transactivation is not critical in the AngII-induced expression changes of the investigated genes. Our data can help us understand the details of AngII-induced long-term effects and the pathophysiology of AT1-R. Moreover, it can help to develop potential interventions for those symptoms that are induced by the over-functioning of this receptor, such as vascular remodeling, cardiac hypertrophy or atherosclerosis.

## 1. Introduction

Angiotensin II (AngII) is an octapeptide hormone that is the main effector of the renin-angiotensin system, and participates in the physiological and pathological mechanisms leading to cardiovascular diseases. The resulting pathophysiological changes, such as vascular remodeling, atherosclerosis and hypertension, are mainly due to the exaggerated action of AngII, which results in hyperplasia and hypertrophy in the cardiovascular tissues [1]. AngII most importantly acts through the type 1 angiotensin II receptor (AT_1_-R), a versatile G protein-coupled receptor (GPCR) that is able to promote a broad variety of biological effects, both short-term and long-term [1,2]. AngII regulates the vascular tone in vascular smooth muscle cells (VSMCs) and it is also an important regulator of cell proliferation and vascular remodeling. The AT_1_-R largely acts via heterotrimeric G_q/11_ activation in VSMCs, resulting in second messenger generation (Ca^2+^ signal via inositol trisphosphate and diacylglycerol) upon agonist binding. This “classical” G_q/11_ protein-mediated signaling mechanism is responsible for the majority of AngII-evoked physiological responses in target cells, but AT_1_-R is able to activate G_i/o_ or G_12/13_ proteins as well [3,4]. In addition, there are other AT_1_-R-mediated signaling mechanisms that are AngII-induced and independent of G protein coupling [5].

AngII stimulation also triggers the activation of receptor tyrosine kinases, among which EGFR transactivation plays the most important role in the cardiovascular system [1]. EGFR transactivation is mediated by matrix metalloprotease activation, which causes the shedding of heparin-binding epidermal growth factor-like growth factor (HB–EGF) resulting in agonist release and binding to EGFR [6,7]. The EGFR transactivation has been proven to be an important factor in the long-term effects of AngII in VSMCs, including cell proliferation. It is also responsible for the development of several pathophysiological conditions in the cardiovascular system, such as vascular remodeling and atherosclerosis [1]. In addition to EGFR transactivation, platelet-derived growth factor receptor and insulin-like growth factor I receptor transactivation appears to be important in cardiovascular cells [1].

The vascular smooth muscle is one of the main targets of AngII. Its stimulation activates numerous signaling pathways that cause contraction and could also result in gene expression changes in VSMCs (Supplementary Figure S1). Although much is understood regarding the mechanisms involved in the regulation of AngII-induced gene expression in various cells [8,9,10,11,12,13], less information is available about the signal transduction pathways involved in primary VSMCs. In this work, we used rat primary isolated VSMCs for our studies to provide more relevant results than we could achieve by studying immortalized cell lines. Although rat primary VSMCs can be maintained up to 20–30 passages, it was demonstrated that primary cultured VSMCs undergo phenotypic modulations [14]. These changes can manifest as early as 7–9 days into primary cell culturing [15]. We carried out the experiments up to 3 passages in order to keep the molecular machinery of the cells as similar to their in vivo conditions as possible.

Mitogen-activated protein kinases (MAPKs), such as ERK1/2, JNK, and p38 MAPK, play an important role in the regulation of various functions in VSMCs and they are general mediators of AngII-evoked cellular responses [16]. The MAPKs require dual phosphorylation of both threonine and tyrosine residues within their activation motif for activation by MAPK kinases. The duration and the magnitude of MAPK activation determine cellular functions such as cell proliferation, gene expression, differentiation, cell death, and metabolism. The activation states of MAPKs are primarily regulated by a family of dual-specificity MAPK phosphatases (DUSPs). DUSPs can dephosphorylate both threonine and tyrosine residues within their activation loop. In addition, they control the duration and the spatiotemporal properties of the MAPK pathways; hence, they are important regulators of MAPK signaling in the cells [17,18]. Interestingly, the binding of DUSPs to MAPKs does not require the phosphorylated, active state of the MAPKs; thus, DUSPs can regulate the availability of various MAPKs and they serve as versatile regulators of MAPK signaling. To date, at least 11 DUSPs have been described in the regulation of activity patterns of MAPKs. DUSPs can be divided into three subfamilies, based on domain structure and subcellular localization [18,19]. DUSP1, DUSP2, DUSP4 and DUSP5 are localized in the nucleus where they can dephosphorylate all three MAPKs, whereas DUSP6, DUSP7 and DUSP9 largely dephosphorylate ERK1/2 MAPK in the cytoplasm. DUSP8, DUSP10, DUSP14 and DUSP16 can be localized in both the nucleus and the cytoplasm, and they mainly regulate the JNK and p38 MAPKs, except for DUSP8, which is more specific for ERK1/2 [20]. The various DUSPs are transiently induced by cellular stresses, or mitogens such as growth factors, and affect the activity of MAPKs. It is well established that their activity and expression are dependent on the regulated MAPKs, thus providing a feedback loop [18].

In the present study, we have sought to investigate the change in transcriptome and identify novel, potentially important but not yet well-characterized proteins that are involved in the action of AngII using primary VSMCs up to their 3 passages. Our transcriptome analysis revealed the upregulation of several DUSP genes, such as *DUSP4*, *5*, *6*, *10*, and *14*. In our further analysis, we have chosen one DUSP gene from each subfamily (based on their intracellular localization), such as *DUSP5*, *DUSP6*, and *DUSP10,* to investigate gene expression changes in response to AngII stimulation.

## 2. Materials and Methods

### 2.1. Materials

Cell culture dishes and plates were purchased from Greiner (Kremsmunster, Austria). Unless otherwise stated, all molecular biology and cell-culture reagents were from Thermo Fisher Scientific (Waltham, MA, USA). Fast Start Essential DNA Green Master Mix was sourced from Roche Applied Science (Basel, Switzerland). The RNeasy Plus Mini Kit was from Qiagen (Hilden, Germany). To maintain cell cultures, Dulbecco’s Modified Eagle Medium (DMEM) was purchased from Biosera (Nuaille, France). Heat-inactivated fetal bovine serum, Glutamax and penicillin/streptomycin were supplied by Invitrogen (Carlsbad, CA, USA). Immobilon Western Chemiluminescent HRP substrate was purchased from Merck-Millipore (Billerica, MA, USA), Radiance Plus Femtogram HRP substrate was obtained from Azure Biosystems (Dublin, CA, USA). Glycerol, sodium dodecyl sulfate (SDS) and 40% acrylamide/bis solution were obtained from Serva (Heidelberg, Germany). Tween 20 and 2-mercapto-ethanol, bromophenol blue, phosphatase inhibitor cocktail 2 were purchased from Sigma-Aldrich (St. Louis, MO, USA). The protease inhibitor cOmplete used in sample preparation for immunoblotting was obtained from Roche Applied Science (Basel, Switzerland). Immunoblot signals were detected with an Azure c600 device (Azure Biosystems, Dublin, CA, USA). The Lenti-X Concentrator kit was from Takara Bio (Kusatsu, Japan). To measure lentivirus concentrations, we used a Lentivirus Titer Kit (Applied Biological Materials, Vancouver, Canada). AngII, EGF, AG1024, AG538, AG1478, BAPTA-AM, CK59, Gefitinib, MMP-2/MMP-9 Inhibitor II, PD98059 and PF 562271 were purchased from Sigma-Aldrich (St. Louis, MO, USA). YM-254890 was obtained from Wako Chemicals (Neuss, Germany). TRV120023 (Sar-Arg-Val-Tyr-Lys-His-Pro-Ala-OH) peptide was synthesized by Proteogenix (Schiltigheim, France) to more than 98% purity. Gefitinib, Sorafenib and Sunitinib were synthesized by Vichem Chemie Research Ltd. (Budapest, Hungary) as members of NCL (Nested Chemical Library). The purity of the compounds was > 99%, determined by LC-MS and NMR. Anti-pERK1/2, anti-pEGFR, anti-mouse-HRP and anti-rabbit-HRP were sourced from Cell Signaling Technologies (Danvers, MA, USA). Anti-α-actin, anti-β-actin antibodies and DAPI (4′,6-diamidino-2-phenylindole, dihydrochloride) were obtained from Sigma-Aldrich (St. Louis, MO, USA). Alexa Fluor 488 conjugated anti-mouse IgG was obtained from Invitrogen (Carlsbad, CA, USA).

The human embryonic kidney (HEK293T) cells were sourced from ATCC (ATCC CRL-3216; American Type Culture Collection, Manassas, VA, USA). Unless otherwise stated, all other chemicals and reagents were purchased from Sigma-Aldrich (St. Louis, MO, USA).

### 2.2. Animals

Male Wistar rats were used for the preparation of primary VSMCs (170–250 g, Charles River Laboratories-Semmelweis University, Budapest, Hungary). They were kept on a standard semisynthetic diet. The animals were then sacrificed by decapitation and rapid bleeding. The investigation conformed to the Guide for the Care and Use of Laboratory Animals (NIH, 8th edition, 2011) as well as to national legal and institutional guidelines for animal care. They were approved by the Animal Care Committee of the Semmelweis University, Budapest, and by the Hungarian authorities (No. 001/2139-4/2012). All procedures followed legal and institutional guidelines of animal care.

### 2.3. Isolation of VSMCs

The rat VSMCs were isolated according to the standard explant method [21]. Briefly, to isolate appropriate amounts of cells for our experiments, over a one-week period, two animals were sacrificed by decapitation and rapid bleeding. After removal of the connective tissue and the adherent fat, the thoracic aorta was excised. The aorta was cut into small sections and the VSMCs were allowed to grow out from the explant for 7–14 days. The VSMCs were maintained by passaging with trypsin and were used between passages 2 and 3 (typically, the experiments were performed at passage 3). We used 4–5 million cells weekly for our experiments. The expression of smooth muscle α-actin was confirmed by immunochemistry (Supplementary Figure S1A). The isolated VSMCs exhibited a normal response to AngII stimulation, such as calcium signals and ERK activation (Supplementary Figure S1B, other data not shown).

### 2.4. Cell Culture

The experiments were conducted on a rat aortic primary isolated VSMC cell line, whereas the lentiviral particles were made using the HEK293T cell line. The cells were subcultured in DMEM supplemented with 10% heat-inactivated fetal bovine serum, 1% Glutamax and 100 IU/mL penicillin/streptomycin, in 5% CO_2_ at 37 °C. For each experiment, VSMCs were transferred onto 6-well plates and were used at approximately 90% confluency. Before the experiments, VSMCs were made quiescent by incubating them in serum-free DMEM for 16–24 h.

### 2.5. Affymetrix GeneChip

After serum deprivation, VSMCs were stimulated with 100 nM AngII for 2 h at 37 °C, then the cells were lysed in Trizol reagent. The quality control of the RNA samples was checked using an Agilent BioAnalyzer RNA Nano lab chip before the array experiments. The total RNA isolation and the Affymetrix Rat Gene 1.0 ST GeneChip Array (Affymetrix, Santa Clara, CA, USA) analysis were performed by UD-GenoMed Medical Genomic Technologies Ltd., University of Debrecen, Debrecen, Hungary). Hybridization and an image scan were performed according to the protocol of UD-GenoMed Medical Genomic Technologies Ltd. The microarray experiment was performed in triplicate. Raw CEL files were background-corrected and normalized using the *oligo* R package [22], and differential expression (Angiotensin II-Vehiculum) analysis was performed using the *limma* R package [22]. We used *PROGENy* pathway activity analysis tool to identify AngII induced pathway activity changes [23,24]. Calculated *PROGENy* pathway activity scores were normalized to null distribution (created by 10,000 random permutations of gene names) to create pathway activity z-scores.

### 2.6. DNA Constructs

For the construction of gene-silencing transfer plasmids, pLKO.1 puro vector was used. This was a gift from Dr. Bob Weinberg (Addgene plasmid #8453; http://n2t.net/addgene:8453; access date: 9 December 2021; RRID: Addgene_8453) [25]. The AgeI and EcoRI restriction sites of the pLKO.1 puro vector and the following sequences transcribing short-hairpin RNAs, specific to rat EGFR, were used: shRNA#1: 5′-GCATAGGCATTGGTGAATTTA-3′ shRNA#2: 5′-GGAAATCACCTATGTGCAAAG-3′ or scrambled sequence (control). The oligos were obtained from Sigma-Aldrich.

### 2.7. RNA Extraction and Real-Time PCR

Cells were washed twice with sterile PBS (137 mM NaCl; 2.7 mM KCl 2.7; 10.1 mM Na_2_HPO_4_; 1.8 mM KH_2_PO_4_, pH 7.4), and the total RNA was isolated with an RNeasy Plus Mini kit from Qiagen. RNA concentrations were determined spectrophotometrically via absorbance at 260 nm and purity was assessed by the 260/280 and 230/260 nm ratios. Reverse transcription from total RNA was carried out using a RevertAid Reverse Transcription Kit according to the manufacturer’s instructions. Gene expression levels were quantified by quantitative real-time PCR (qRT-PCR). The measurements were performed using the SYBR Green method (SYBR Green I Master, Roche, Basel, Switzerland) using a LightCycler 480. The primers were synthesized by Sigma-Aldrich and designed so that the amplicon sizes were between 100 and 200 base pairs. Efficiency for each primer pair was determined by using serial dilutions of the PCR product.

The thermal cycling program started with pre-incubation at 95 °C for 5 min, followed by amplification via 45 cycles of 10 s at 95 °C, 5 s at 62 °C and 15 s at 72 °C, melting curve 5 s at 95 °C, 1 min at 65 °C and 97 °C, and cooling for 30 s at 40 °C. Fluorescence data including melting curves were obtained. The cycle threshold (Ct) was calculated via the second derivative method using LightCycler 480 Software. ΔCt represents the difference in Ct values obtained between the reference and the tested samples. For normalization, the glyceraldehyde-3-phosphate dehydrogenase (*GAPDH*) housekeeping gene was used. Gene expression levels were plotted against the *GAPDH* expression level. Fold ratios of gene expression were calculated as follows: ratio = E^ΔCt target gene^/E^ΔCt GAPDH^. The following primers were used for qRT-PCR determinations (5′-3′): GAPDH: Forward CCT GCA CCA CCA ACT GCT TAG, Reverse CAG TCT TCT GAG TGG CAG TGA TG; DUSP5: Forward GGC AAG GTC CTG GTT CAC TGT, Reverse GTT GGG AGA GAC CAC GCT CCT; DUSP6: Forward ATC ACT GGA GCC AAA ACC TG, Reverse CGT TCA TGG ACA AGT TGA GC; DUSP10 Forward GGC AAA GAA CCC CTG GTA TT, Reverse AGA AAC AGG AAG GGC AGG AT; EGFR Forward CAT CCA GTG CCA TCC AGA AT, Reverse CTT CCA GAC CAG GGT GTT GT.

### 2.8. Treatment Protocols

Before the experiments, VSMCs were made quiescent by incubating in serum-free DMEM for 16–24 h. For the time-dependency determinations, the serum-starved cells were stimulated with 100 nM AngII for 1–6 h. During the examination of inhibitor effects, the cells were pretreated with the appropriate inhibitor for 30 min, then stimulated for 2 h with 100 nM AngII or 50 ng/mL EGF. BAPTA-AM and RO31-8425 pretreatments were used for 10 min before the 2-h agonist stimulation of the cells.

### 2.9. Lentivirus Production

Lentiviruses were produced by co-transfecting HEK293T cells on 10-cm dishes with pLKO.1puro transfer, the pCMV-VSV-G envelope and pCMV-dR8.2 packaging plasmids (gift from Dr. Bob Weinberg, [25], purchased from Addgene) using the calcium phosphate precipitation method. In summary, plasmid DNAs were mixed in sterile distilled water, then 2.5 M CaCl2 was added (final concentration: 125 mM) and the solution was mixed dropwise with 2× HEPES-buffered solution [HBS] (42 mM HEPES, 15 mM D-glucose, 1.4 mM Na2HPO4, 10 mM KCl, 274 mM NaCl 274 mM, pH 7.1). This mixture was added dropwise to attached cells and the medium was replaced with fresh complete DMEM after 6 h. After 48 h had passed post-transfection, the cell medium was collected and centrifuged for 10 min at 3000 rpm, the supernatant was filtered and the lentiviral vector particles were purified and concentrated with a Lenti-X concentrator kit (Takara). After concentration, the viral particles were resuspended in sterile phosphate-buffered saline and the titer of the samples was measured with a qPCR Lentivirus titer kit from Applied Biological Materials (Vancouver, Canada). The samples were stored at −80 °C until the infection of cells.

### 2.10. Lentiviral Infection of VSMCs

Briefly, 2 × 10^5^–2.5 × 10^5^ primary vascular smooth muscle cells/well were plated on-6-well plates, and the cells were infected the next day. The same titers of lentiviral preparations, diluted in complete DMEM + 8 µg/mL Polybrene (Sigma-Aldrich), were used to infect the VSMCs. The cells were subjected to the experiments 48 h after infection.

### 2.11. Immunoblot Analysis

After the agonist stimulation of the VSMCs, cells were scraped with 2 × concentrated Laemmli buffer (Tris-Cl pH 6.8, glycerol, SDS, 2-mercapto-ethanol, bromophenol blue), supplemented with protease and phosphatase inhibitors. This cell lysate was briefly sonicated, then boiled, and equal amounts of samples were loaded into 12% SDS-polyacrylamide gels. The proteins were transferred to PVDF membranes using 80V for 2 h during the process. The PVDF membranes were then blocked with a 5% blocking solution (nonfat dried milk diluted in PBS-T). The membranes were then incubated with either pEGFR or pERK1/2 primary antibodies, depending on the experiment. Following PBS-T washing, these membranes were incubated with the appropriate anti-rabbit IgG secondary antibody. As a loading control, β-actin labeling was used. The signals were visualized with enhanced chemiluminescence, using Immobilon Western HRP substrate reagents, and were then detected with an Azure c600 device.

### 2.12. Immunofluorescence Staining

The immunostaining was performed according to the following protocol: the VSMCs were gently washed, once, before fixation with 3.7% paraformaldehyde solution for 15 min. After quenching the fixation solution in three washing steps, the cells were permeabilized using 0.1% Triton X-100 (Sigma-Aldrich) and incubated in 0.1% sodium-borohydride solution for 15 min. The cells were then incubated in 1% BSA (Sigma-Aldrich) containing blocking solution for 30 min, then immunolabelled using anti-α-smooth muscle actin (Sigma-Aldrich) and Alexa Fluor 488 conjugated secondary antibodies (Invitrogen). Cell nuclei were stained by DAPI (4′,6-diamidino-2-phenylindole, dihydrochloride; Sigma-Aldrich). Photomicrographs were taken using a Leica DMI6000B inverted microscope.

### 2.13. Statistical Analysis

We analyzed gene expression data, collected from qRT-PCR measurements, using multiple linear regression with a 95% confidence interval in order to determine the significance of inhibitor treatments, stimuli and their interaction on the dependent variable, which is the fold-change value of a given gene of interest. In the case of Figures 2, 4D–F and 6A, ordinary one-way ANOVA analyses were performed to compare stimulated or lentiviral infected groups to control groups. Statistical analysis and graph plotting were carried out with GraphPad Prism 9.1.2 software. The sample size (*n*) in the figure legends refers to the number of independent experiments (biological replicates). Unless otherwise stated, data are presented as mean ± SE.

## 3. Results

### 3.1. Affymetrix GeneChip Analysis of the AngII Upregulated Genes in VSMCs

In the present study, we used the Affymetrix GeneChip Rat Gene 1.0 ST array to compare the gene expression profiles of the vehicle and 100 nM AngII-treated VSMCs after 2 h of treatment. We used rat primary isolated VSMCs in their second passage, in order to get physiologically relevant data. In order to demonstrate that these relatively early-passage young cells possess the expected properties of VSMCs, we performed smooth muscle α-actin immunostaining and agonist stimulation of these early passage cells. Our data confirmed that these cells showed characteristic features of VSMCs, such as the expression of smooth muscle α-actin and showed a typical ERK1/2 activation pattern in response to 50 ng/mL EGF or 100 nM AngII stimulation (Supplementary Figure S1).

**Figure 1:**
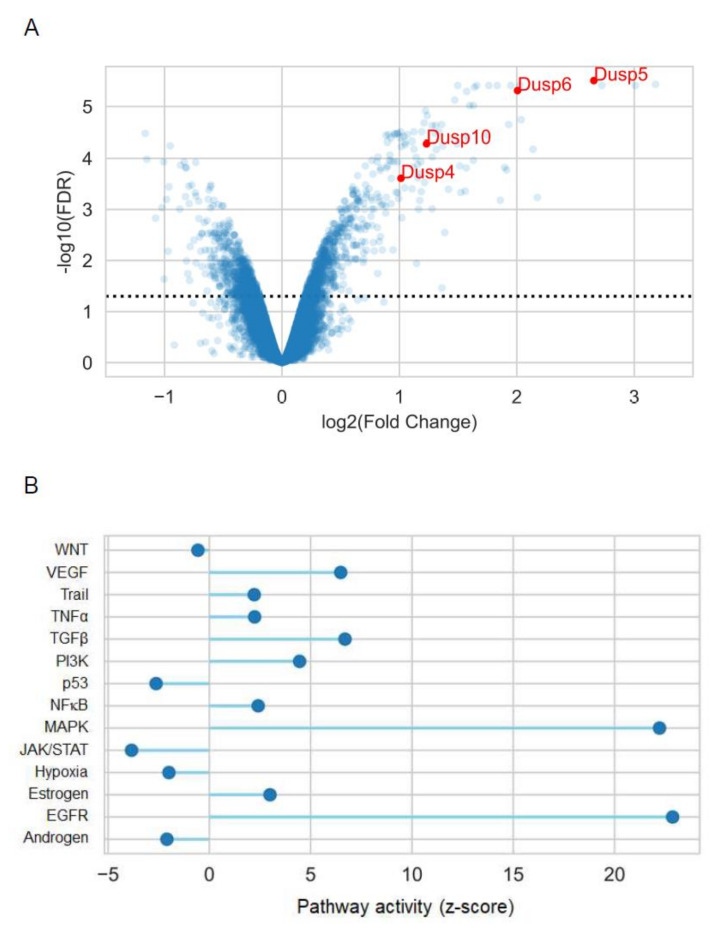
AngII-induced gene expression changes in VSMCs. (**A**) Differential expression (DE) analysis was performed between 2-hour AngII- and vehicle-treated samples. The microarray experiment was performed in triplicate. The results of DE analysis are shown as a volcano plot (*x*-axis: log2 fold change, *y*-axis: −log10 (FDR, false discovery rate), based on the Benjamini–Hochberg correction of p-values. Selected members of the DUSP family are color-coded and text-labeled. The dotted line shows the significance threshold (FDR < 0.05) (**B**) *PROGENy* pathway analysis of an AngII-induced gene expression signature. Pathway activity was calculated for 14 PROGENy pathways (*x*-axis) and normalized to z-scores (*y*-axis), based on a random permutation of gene labels.

We performed differential expression (DE) analysis between the AngII- and vehicle-treated microarray samples using *limma* [21]. AngII led to the significant upregulation (false discovery rate, based on the Benjamini–Hochberg correction < 0.05, log_2_ fold change > 1) of 74 genes (Figure 1A). We found 4 DUSP isoforms among the significantly upregulated genes, namely, DUSP4, DUSP5, DUSP6 and DUSP10 (log2 fold change values: 1.01, 2.66, 2.01 and 1.23, FDR (false discovery rate): 2.5 × 10^−04^, 3 × 10^−06^, 5 × 10^−06^ and 5.2 × 10^−05^, respectively). We performed a pathway activity analysis using the *PROGENy* tool to ensure a more unbiased analysis of AngII-induced gene expression changes [22,23]. *PROGENy* identifies upstream pathways regulating the observed gene expression changes for 14 pathways (Figure 1B). *PROGENy* analysis revealed that AngII treatment significantly increased the MAPK and EGFR pathways (Figure 1B, z-scores of pathway activities: 21.22 and 22.86, respectively (see the Materials and Methods section for further details)), and also led to a modest increase in another receptor tyrosine kinase pathway (VEGFR) and TGFβ.

In summary, our microarray analysis revealed that AngII increased the activity of MAPK and EGFR pathways in VSMCs. We found several DUSP isoforms, important negative regulators of the MAPK pathway, among the most significantly overexpressed genes, corresponding to a plausible negative feedback mechanism [26].

### 3.2. qRT-PCR Measurements Validate the Affymetrix Array Results Regarding the Upregulation of DUSP5, DUSP6, and DUSP10 Gene Expressions, in Response to AngII Stimulation. Time Kinetics of Gene Expression Changes, in Response to the AngII Stimulation of VSMCs

**Figure 2:**
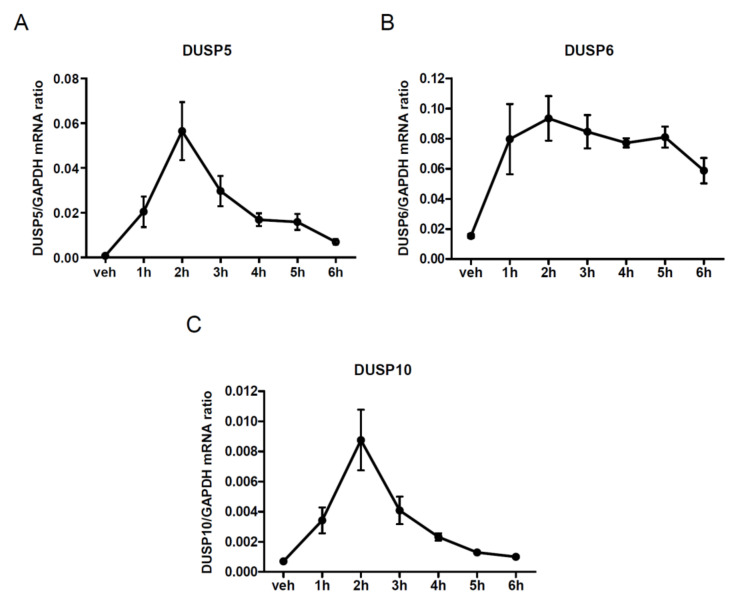
Time-dependent effect of AngII stimulation on gene expression in VSMCs. VSMCs were serum-depleted for 24 h, then cells were treated for various lengths of time intervals with 100 nM AngII, beside a control group treated with the vehicle. Time kinetics of (**A**) DUSP5, (**B**) DUSP6, and (**C**) DUSP10 expression levels are shown. The mRNA abundance was calculated via normalization to the *GAPDH* housekeeping gene and measured using real-time PCR. Mean values ± SE are shown (*n* = 5–6).

We used qRT-PCR determinations to confirm the effect of AngII stimulation on the expression levels of certain DUSP isoforms. The transcriptome analysis revealed the upregulation of several DUSP genes, such as *DUSP 5*, *6*, *10*, *4*, and *14*. We selected one DUSP from each subfamily of DUSPs, namely, DUSP5, DUSP6, and DUSP10 for our studies. We aimed to validate the Affymetrix GeneChip results, and we also wanted to determine the time course of the AngII-induced DUSP5 (Figure 2A), DUSP6 (Figure 2B) and DUSP10 (Figure 2C) expression-level upregulation. VSMCs were stimulated at different points of time from 1 to 6 h with 100 nM of AngII, then the mRNA levels were measured via real-time PCR. In the case of DUSP5 and DUSP10, we observed the highest mRNA levels 2 h after the stimulation. The mRNA levels of DUSP6 were strongly elevated after the first hour, peaked at 2 h, and remained continuously elevated, although they showed a slightly reduced tendency at later time points. Based on the qRT-PCR results, we chose 2-hour-long stimulations for our further experiments concerning the analysis of gene expression changes.

### 3.3. Investigation of Signaling Pathways Involved in AngII-Mediated Responses

**Figure 3:**
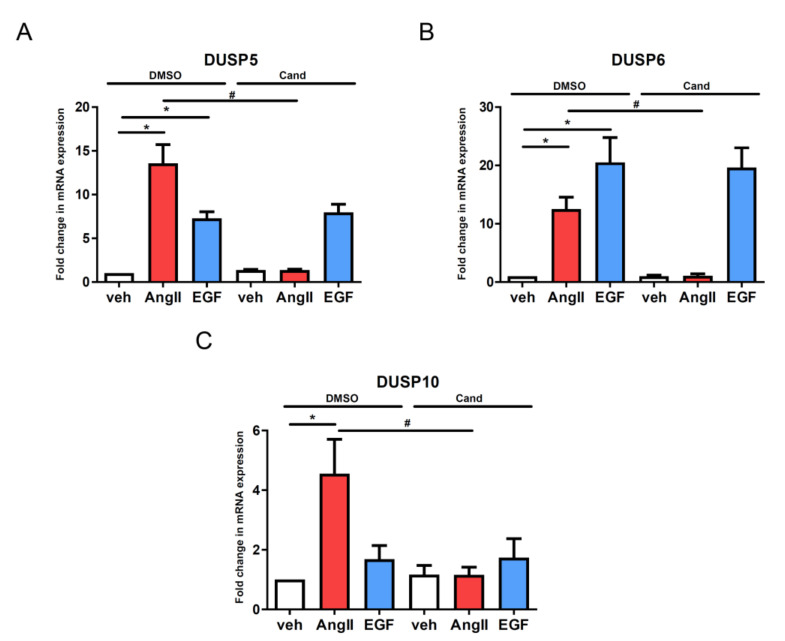
Effect of AT_1_-R antagonist treatment on the agonist-induced gene expression changes of the DUSP isoform in vascular smooth muscle cells. Serum-starved cells were incubated with 10 µM candesartan (Cand) or DMSO as a control for 30 min, then the cells were exposed to either 100 nM AngII (red columns) or 50 ng/mL EGF (blue columns) or the vehicle (white columns) for 2 h. Standardization was made against the *GAPDH* housekeeping gene. The mRNA levels of DUSP5 (**A**), DUSP6 (**B**) and DUSP10 (**C**) were normalized to values of DMSO vehicle samples and expressed as fold change. Mean values ± SE are shown. Significance was determined with multiple linear regression. *p* < 0.05 was considered as statistically significant. *: statistically significant from vehicle stimulation. #: statistically significant from DMSO-pretreated agonist-induced response. The values are from four independent experiments (*n* = 4).

Next, we wanted to determine which receptor type and coupling G protein is responsible for the expression changes. Theoretically, AngII can stimulate and mediate its effect via two distinct GPCRs, the AT_1_ and AT_2_ angiotensin receptors in VSMCs [27]. The AT_1_-R is much more important and is abundantly expressed in vessels. In order to exclude the potential role of the AT_2_ angiotensin receptor in the investigated gene expression changes, we applied candesartan, a selective AT_1_-R antagonist with insurmountable binding properties. As shown in Figure 3, the candesartan (10 µM) pretreatment completely blocked the AngII-mediated upregulation of DUSP levels. This data indicated that AT_1_-R mediates the observed AngII-induced gene expression changes that mostly couple to the G_q/11_ heterotrimeric G protein in VSMCs [1].

**Figure 4:**
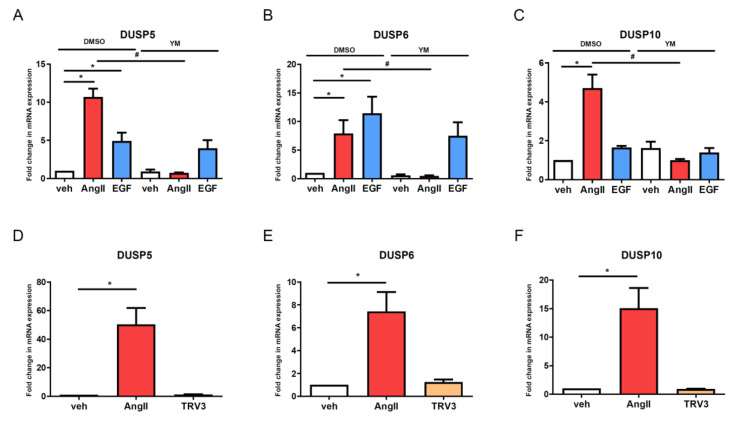
Evaluation of the contribution of G-protein-dependent and independent mechanisms in the AT_1_-receptor stimulation on induced changes in DUSP levels in vascular smooth muscle cells. Serum-depleted VSMCs were incubated with 1 µM YM-254890 (YM) or DMSO as a control for 30 min, then the cells were exposed to either 100 nM AngII (red columns) or 50 ng/mL EGF (blue columns) or vehicle (white columns) for 2 h (**A**–**C**). The serum-starved VSMCs were exposed to either the vehicle (white columns) or 100 nM AngII I (red columns) or 3 µM TRV120023 (beige columns) for 2 h (**D**–**F**). RNA was isolated from VSMCs, then converted to cDNA. cDNA levels of DUSP5 (**A**,**D**), DUSP6 (**B**,**E**) and DUSP10 (**C**,**F**) were measured by qRT-PCR. Standardization was established against the *GAPDH* housekeeping gene. Mean values ± SE are shown. Significance was determined via multiple linear regressions. *p* < 0.05 was considered as statistically significant. *: statistically significant from vehicle stimulation. #: statistically significant from DMSO pretreated agonist-induced response (**A**–**C**). In the case of **D**–**F,** significance was determined with a one-way ANOVA-test (* *p* < 0.05). The values are from four or five independent experiments (*n* = 4–5).

It has previously been reported that only the G protein-dependent mechanism of AT_1_-R seems to be important in AngII-induced hypertrophy in VSMCs [28]. In addition to the G_q/11_ activation mechanism, it is well documented that AT_1_-R can also couple to the G_i/o_ and G_12/13_ heterotrimeric proteins, leading to the inhibition of adenylyl cyclase, the activation of Rho-kinase and phospholipase D, and the regulation of Ca^2+^ channels [29]. We used YM-254890, a selective G_q/11_ inhibitor, to investigate the role of G_q/11_-mediated pathways. Figure 4 demonstrates that 1 µM of YM-254890 completely wiped out the AngII-mediated gene expression upregulation in all the examined DUSPs. We also used pertussis toxin (PTX) pretreatment (100 ng/mL for 18 h) to inhibit G_i_ protein activation and to assess the potential role of the G_i_ protein in the induced gene expression changes. Our results show that the PTX evoked only partial inhibition of the AngII-induced gene expression changes, emphasizing the primary role of G_q/11_ activation (data not shown). We also wanted to evaluate the possible role of β-arrestin-mediated signaling in the regulation of AngII-induced expression changes. TRV120023 peptide is a biased agonist of AT_1_-R that triggers no or partial activation of G proteins but it induces β-arrestin-mediated signaling via β-arrestin binding [30,31,32]. In contrast to 100 nM AngII stimulation, using 3 µM TRV120023 as an AT_1_-R agonist did not evoke significant gene expression changes, which reflects the finding that DUSP upregulation is initiated exclusively in a G-protein-dependent manner (Figure 4).

### 3.4. Effect of EGF Stimulation and EGFR Tyrosine Kinase Inhibitors on Agonist-Induced Expression of DUSP Genes

**Figure 5:**
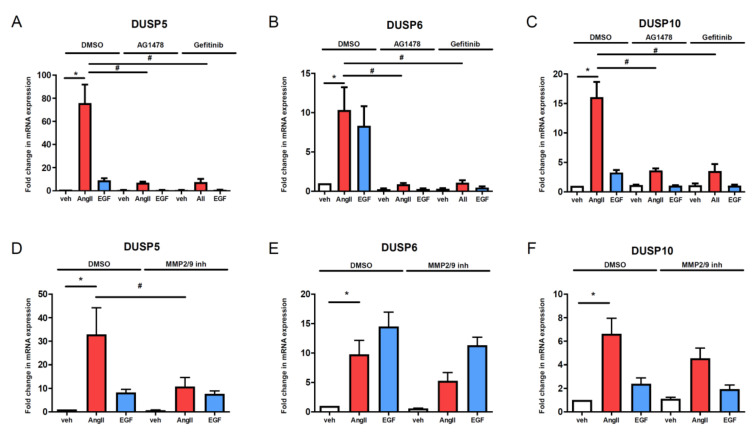
Effect of EGFR transactivation on the gene expressional changes of DUSP isoforms in VSMCs. Serum starved cells were incubated with either 1 µM AG1478 (**A**–**C**) or 2.5 µM gefitinib (**A**–**C**), or 1 µM MMP-2/MMP-9 Inhibitor II (**D**–**F**) beside the control treated with DMSO for 30 min, then the cells were exposed to either 100 nM AngII (red columns) or 50 ng/mL EGF (blue columns) or vehicle (white columns) for 2 h. mRNA levels of DUSP5 (**A**,**D**), DUSP6 (**B**,**E**) and DUSP10 (**C**,**F**) were measured by qPCR. Standardization was made against the *GAPDH* housekeeping gene. The mRNA levels were normalized to values of DMSO vehicle samples and expressed as fold change. The values are from three-six independent experiments. Mean values ± S.E. are shown. Significance was determined with multiple linear regression. *p* < 0.05 was considered as statistically significant. *: statistically significant from vehicle stimulation. #: statistically significant from DMSO-pretreated agonist-induced response. The values are from three to six independent experiments (*n* = 3–6).

Since EGF-receptor transactivation plays an important role in the AngII-induced cell responses in VSMCs, we investigated the effect of direct EGF-receptor stimulation on DUSP gene-expression changes. Figure 5 demonstrates that 50 ng/mL of EGF stimulation caused a significant increase in DUSP5 and DUSP10 expression levels but this was to a lesser extent than the 100 nM AngII stimulus-evoked response (Figure 5A,C). In the case of DUSP6 mRNA levels, we observed a similar increase in both EGF- and AngII-stimulated groups (Figure 5B). In order to determine the role and contribution of EGFR transactivation in the AngII-induced changes, we pretreated the VSMCs with either 1 µM AG1478 or 2.5 µM gefitinib, two widely used EGFR tyrosine kinase inhibitors. As expected, these inhibitors completely blocked the EGF-induced increase of DUSP5, DUSP6, and DUSP10 mRNA levels (Figure 5). It is noteworthy that neither candesartan nor YM-254890 evoked a significant effect on the EGF-induced increase in DUSP expression levels, showing the specificity of the candesartan and YM-254890 on AngII-induced responses (Figure 3 and Figure 4A–C). It is important to note that although the AngII-induced upregulation of DUSP levels was significantly blunted by AG1478 and gefitinib pretreatment, these inhibitory effects were not total.

### 3.5. Inhibition of Matrix Metalloproteinases Has a Moderate Effect on AngII-Induced DUSP Upregulation

It is well documented that the matrix metalloproteinases are key mediators of AngII-induced transactivation of EGFR in VSMCs and are important effectors of AngII-mediated vascular remodeling [1,6,7]. Among the MMPs, MMP-2 and MMP-9 appear to play the most important role in cardiovascular cells and, using a highly selective inhibitor, MMP-2/MMP-9 Inhibitor II potently inhibited EGFR transactivation in VSMCs [7,33]. As expected, the MMP-2/MMP-9 Inhibitor II had no significant effect on direct EGFR activation-induced gene expression changes in DUSP genes (Figure 5D–F, blue columns). On the other hand, the MMP-2/MMP-9 Inhibitor II reduced the AngII-induced increase in DUSP5 but the inhibitory effect was not complete (Figure 5D, red column). Moreover, the MMP-2/MMP-9 Inhibitor II had no significant effect on AngII-induced DUSP6 and DUSP10 upregulations (Figure 5E,F, red columns).

### 3.6. The Role of Calcium Signaling and Calcium-Dependent Kinases in the Upregulation of DUSP Levels

The stimulation of AT1 and EGF receptors induces a cytosolic calcium-level increase via the phospholipase β and γ activation mechanisms [1,34,35]. The agonist-induced calcium signal initiates several important regulatory mechanisms, such as the activation of protein kinase C, proline-rich tyrosine kinase 2 (Pyk2) and calcium/calmodulin-dependent protein kinase in VSMCs [36,37,38]. We used 50 μM BAPTA-AM (a permeant calcium chelator) pretreatment prior to agonist stimulation, to investigate the role of intracellular calcium in the regulation of DUSP mRNA levels. The results of the calcium chelation by BAPTA-AM demonstrate that the induced intracellular calcium elevation is essential for the observed upregulation of *DUSP* genes in response to both AngII and EGF stimulations in VSMCs (Supplementary Figure S2A–C). In order to further explore calcium signal-related mechanisms, we applied a specific calcium/calmodulin-dependent protein kinase II (CaMKII) inhibitor, CK59. CaMKII plays an important role in AngII-induced vascular reactivity, hypertrophy in VSMCs, and vascular remodeling [39,40]. Supplementary Figure S2D–F illustrates that the inhibition of CaMKII with 50 μM CK59 significantly attenuated the AngII-induced DUSP5 mRNA level increase but not the response caused by EGF. In the case of DUSP6 and DUSP10, the CK59 also significantly reduced the AngII-induced gene expression upregulations but the effect was much lower in the EGF-stimulated cells (Supplementary Figure S2D–F). To evaluate the role of the calcium-dependent protein kinase C (PKC), we employed the RO31-8425 compound, which is a highly selective inhibitor of PKC [41]. Pretreatment with the RO31-8425 PKC inhibitor (1 μM for 10 min) reduced the AngII- but not the EGFR-induced DUSP5 mRNA level increase (Supplementary Figure S3A–C). The inhibition of PKC resulted in a greatly reduced effect of AngII in DUSP6 and DUSP10 upregulation, whereas EGF-induced effects were moderately inhibited (Supplementary Figure S3A–C). Next, we investigated the involvement of Pyk2, a regulator of EGFR transactivation in VSMCs. We assessed the role of Pyk2 in the AngII induced upregulation of DUSPs by applying PF-562271, a potent ATP-competitive FAK and Pyk2 kinase inhibitor. Pretreatment of the cells with 1 μM PF-562271 slightly but not significantly reduced the AngII-induced upregulation of DUSP5 and DUSP6 (Supplementary Figure S3D,E); the PF-562271 pretreatment did not influence the AngII-mediated gene expressional increase of DUSP10 either (Supplementary Figure S3F).

### 3.7. Silencing of EGFR in VSMCs Using a Lentiviral shRNA System

**Figure 6:**
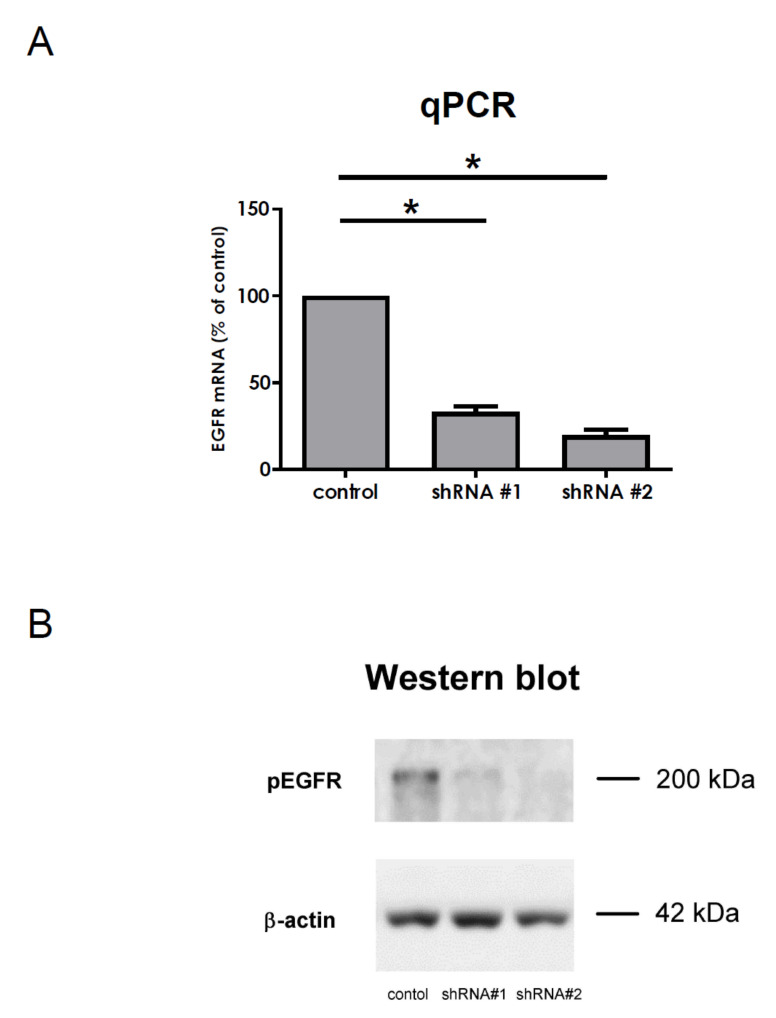
Silencing EGFR expression with shRNA constructs via lentiviral infection. VSMCs were infected with either shEGFR#1 or shEGFR#2 lentiviral constructs for 48 h. Control-group cells were infected with a lentivirus coding-scrambled shRNA sequence. (**A**) *EGFR* gene expression levels were measured by a real-time PCR. Mean values ± SE are shown. Significance was calculated with an ordinary one-way ANOVA test (* *p* < 0.05). (**B**) In the image, 5 min of 50 ng/mL EGF stimulation-induced phospho-EGFR levels were measured by Western blot analysis. Anti-β-actin staining was used as a loading control. The Western blots shown are representative of three independent experiments (*n* = 3).

AngII stimulation can result in the much higher upregulation of DUSP5 and DUSP10 mRNA levels than direct EGFR stimulation by EGF (i.e., Figure 3A,B, red vs blue solid columns). The relative ineffectiveness of matrix metalloproteinase inhibition (Figure 5D–F) raised the possibility that AngII-induced DUSP upregulation is not entirely EGFR-transactivation-dependent. The effects of EGFR inhibitors (AG1478 and gefitinib in Figure 5A–C) on AngII-induced changes may reflect the off-target effects of these inhibitors [42,43,44]. In order to check the role of EGFR in AngII-induced DUSP upregulations, we decided to use the short hairpin RNA (shRNA)-based silencing of EGFR expression via RNA interference. As primary VSMC cultures are hard to transfect conventionally, we transduced VSMCs with pLKO.1 puro lentiviral constructs to produce siRNA specific to EGFR. First, we prepared two sets of lentiviruses containing different constructs. Following infection, we examined *EGFR* mRNA levels via real-time PCR (Figure 6A) and EGFR protein levels via Western blot analysis (Figure 6B). The results indicate that both constructs successfully reduced the expression and the protein levels of EGFR. Briefly, shEGFR#2 was slightly more effective; therefore, we used this in our next experiments.

**Figure 7:**
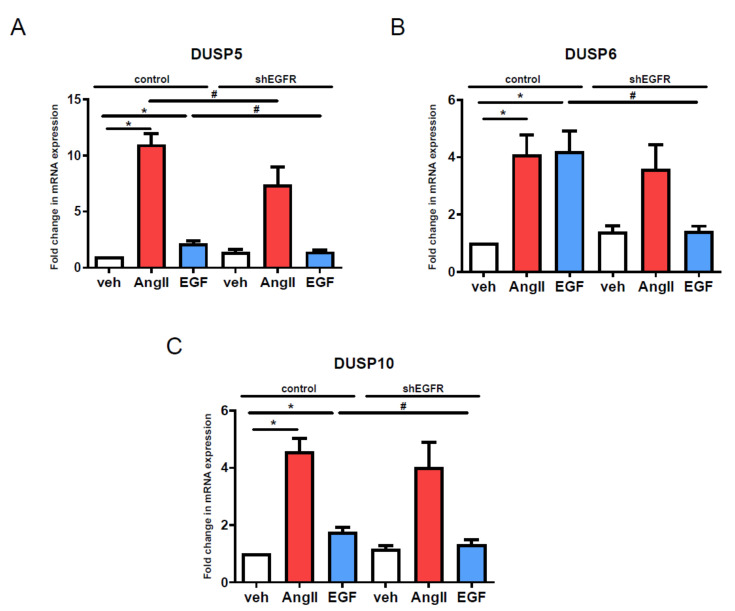
Effects of EGFR silencing on the gene expression responses to AngII and EGF stimuli in VSMCs. Primary vascular smooth muscle cells were infected with either scrambled (control) or shEGFR#2 (shEGFR) shRNA coding lentiviral particles for 48 h. Infected cells were stimulated for 2 h with either 100 nM AngII (red columns), or 50 ng/mL EGF (blue columns) or vehicle (white columns) after 24 h of serum starvation. RNA was isolated from VSMCs, then converted to cDNA. cDNA levels of DUSP5 (**A**), DUSP6 (**B**) and DUSP10 (**C**) were measured by qRT-PCR. Standardization was made against the *GAPDH* housekeeping gene. The mRNA levels were normalized to values of control virus-infected and vehicle-stimulated samples and expressed as fold change. Mean values ± S.E. are shown. Significance was determined with multiple linear regression. *p* < 0.05 was considered as statistically significant. *: Statistically significant from vehicle stimulation. #: Statistically significant from control virus-infected agonist-induced response. The values are from four independent experiments (*n* = 4).

The results shown in Figure 7 demonstrate that the shRNA silencing of EGFR has less effect on AngII-mediated gene expression changes than pharmacological inhibition. After waiting for 48 h after lentiviral infection with shRNA coding particles, VSMCs were stimulated with AngII or EGF, apart from a control group treated with the vehicle. The mRNA levels of DUSP isoforms were measured by real-time PCR. As expected, the silencing of EGFR completely wiped out the EGF stimulation-mediated DUSP level upregulations (Figure 7, blue columns). In contrast, the gene silencing of EGFR did not lead to such a dramatic blockade in the AngII-induced increase in DUSP mRNA levels, compared to the effect of AG1478 and gefitinib (Figure 7, red columns vs. Figure 5A–C, red columns). Moreover, the silencing of EGFR only caused a significant effect regarding the AngII-induced DUSP5 mRNA increase (Figure 7A, red columns) but in the case of DUSP6 and DUSP10, the EGFR silencing did not cause statistically significant effects (Figure 7B,C, red columns).

### 3.8. Role of Other Growth Factor Receptor Transactivation Mechanisms in the AngII-Induced Gene Expression Changes

Although EGFR transactivation is considered to be the major growth factor receptor transactivation mechanism in VSMCs, the stimulation of AT_1_-R has also been confirmed to transactivate other growth factor receptors, including the platelet-derived growth factor receptor (PDGFR) and the insulin-like growth factor I receptor (IGF-IR) [45,46]. Inhibition of PDGFR with sunitinib or sorafenib pretreatments (Supplementary Figure S4A–C) and inhibition of IGF-IR with AG1024 or AG538 pretreatments (Supplementary Figure S4D–F) did not cause such robust effects as EGFR inhibition with AG1478 or gefitinib (Figure 5A–C) on AngII-induced DUSP expression levels. Only the sorafenib caused a significant reduction in AngII-induced gene expression changes, in the case of *DUSP10,* among these investigated drugs (Supplementary Figure S4C). These results indicate that PDGFR or IGF-IR transactivation is not a major signaling route to lead to AngII-induced upregulation of the investigated DUSP genes.

### 3.9. Effect of Simultaneous AngII and EGF Stimuli on DUSP Levels in VSMCs

**Figure 8:**
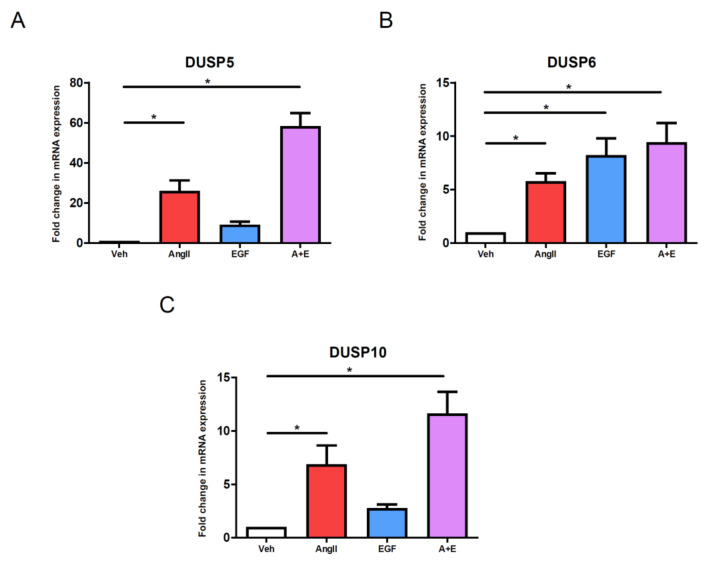
DUSP expression changes after simultaneous AngII and EGF stimuli in VSMCs. Serum-starved cells were exposed to either vehicle (white columns), or 100 nM AngII (red columns), or 50 ng/mL EGF (blue columns), or simultaneously 100 nM AngII and 50 ng/mL EGF (purple columns) for 2 h. RNA was isolated from VSMCs, then converted to cDNA. cDNA levels of DUSP5 (**A**), DUSP6 (**B**) and DUSP10 (**C**) were measured by qRT-PCR. The mRNA levels were normalized to values of DMSO vehicle samples and expressed as a fold change. Mean values ± SE are shown. Significance was determined with multiple linear regression (* *p* < 0.05). The values are from seven independent experiments (*n* = 7).

We used a different approach to confirm that AngII-induced gene expression changes are not exclusively EGFR transactivation-dependent. We stimulated the AT_1_-R and the EGFR together by applying simultaneous 100 nM AngII and 50 ng/mL EGF stimuli of vascular smooth muscle cells. Surprisingly, the DUSP5 level was robustly upregulated. The DUSP5 expression level increased ~2.24-fold in response to simultaneous AngII and EGF stimuli compared to single AngII stimulation, and ~6.38-fold compared to only EGF stimulation (Figure 8A). Although the simultaneous AngII and EGF stimulations caused the highest increase in the DUSP6 level (Figure 8B), the combined effect of receptor agonists was not so striking as in the case of DUSP5. Similar to the evoked effect on DUSP5 level by simultaneous receptor activations, the DUSP10 mRNA level was also significantly upregulated by combined AngII and EGF stimulations; the DUSP10 expression level increased ~1.69-fold compared to only AngII stimulation, and ~4.17-fold compared to only EGF stimulation (Figure 8C). These results clearly demonstrate that there are synergistic pathways that may amplify each other, leading to robust gene expression changes in VSMCs, as in the case of DUSP5 and DUSP10 expression levels.

### 3.10. Effect of MAPK Signaling Inhibition on AngII-Induced DUSP Gene Upregulations

Since the activity and expression of various DUSP isoforms are dependent on MAPKs [18], we aimed to evaluate the role of MAPKs in AngII-induced DUSP expression changes. We pretreated the cells with the PD98059 MEK inhibitor to assess the potential role of ERK1/2 MAPKs in the AngII-evoked responses. The inhibition of the MEK significantly reduced the DUSP5 mRNA level increase upon AngII stimulation (Supplementary Figure S5A). Due to the dramatic effect of the PD98059 MEK inhibitor on basal DUSP6 and DUSP10 levels, the role of ERK1/2 MAPKs in AngII-induced DUSP6 and DUSP10 changes cannot be clearly established (Supplementary Figure S5B,C). On the contrary, the p38 MAPK inhibitor (SB202190) effectively reduced all the investigated AngII stimulation-caused DUSP mRNA level increases in VSMCs, whereas its inactive analog, SB202474, had no effect (Supplementary Figure S5D–F). These results indicate the important role of p38 MAPK signaling in the regulation of expression levels of DUSP5, DUSP6 and DUSP10 isoforms.

## 4. Discussion

Sustained AngII actions can lead to hypertension, vascular remodeling and atherosclerosis [1]; therefore, an evaluation of AngII-induced gene expression changes is important to clarify. New data can reveal previously unidentified mechanisms and new therapeutic agents to ameliorate AngII-triggered cardiovascular symptoms. Our results provide novel insights into the transcriptomic effects of AngII in primary rat VSMCs. The major findings of the present study are: (1) analyzing the transcriptomic effects and the gene expression changes of various DUSP isoforms in response to AngII stimulations of primary vascular smooth muscle cells and (2) demonstrating that AT_1_-R and EGFR can initiate synergistic signaling pathways to induce gene expression changes. These findings support the notion that AT_1_-R is capable of activating multiple signaling pathways that may be responsible for various cell responses.

Recent studies have already elucidated the importance of AngII-regulated gene expression changes in many cell types [8,9,10,11,12,13]. In this present study, we used rat primary VSMCs up to 3 passages in order to approximate them more closely to their physiological functions. We investigated the transcriptomic effect of AngII by stimulating serum-deprived early-passage VSMCs with the vehicle or 100 nM AngII for two hours. We found many genes that were significantly upregulated or downregulated after AngII stimulation by using the Affymetrix GeneChip assay (Figure 1). In agreement with the previous gene-chip results, numerous earlier-described genes were identified in VSMCs [9,10]. The activation of AT_1_-R is followed by events leading to the downregulation of AT_1_-R; in addition, the initiated signal transduction steps can be attenuated by various mechanisms, i.e., the phosphorylated proteins undergo dephosphorylation reactions. Our data demonstrate that Ang-II upregulates numerous genes, including dual-specificity phosphatases (DUSPs), which, in turn, can regulate the long-term MAPK signaling mechanisms of AT_1_-R. Since the effect of AngII on DUSP gene expressions has not been reported, we wanted to investigate their roles in AngII signaling, and to identify which arm of the AT_1_-R signaling pathway is responsible for these effects. Among the identified genes, we have chosen to analyze one member from each subfamily of DUSP isoforms, namely, DUSP5, DUSP6 and DUSP10. Real-time PCR measurements demonstrated that the expression levels of the investigated DUSP genes increased 1 h after AngII stimulation, peaked at 2 h and persisted for up to 6 h (Figure 2). In the case of DUSP6, the kinetics of the expression were slightly different from those of DUSP5 and DUSP10, which indicates that the involved signaling pathways may vary in terms of the different AngII-evoked responses. We analyzed the possible signal transduction mechanisms that are responsible for the upregulation of the DUSP genes, in response to the AngII stimulation of VSMCs. The results revealed that the AngII effects are due to AT_1_-R activation caused by G_q/11_ protein coupling (Figure 4A–C). The activated AT_1_-Rs interact with β-arrestins that serve as organizers/scaffold platforms of signaling complexes, such as the activation of the MAP kinase cascade [47]. In order to evaluate the possible role of β-arrestin-mediated signaling in the regulation of expression changes, we applied TRV120023, a β-arrestin-biased AT_1_-R agonist, and the results indicated that β-arrestin has no significant role in the regulation of DUSP expression levels (Figure 4D–F). Since the G_q_-dependent signaling of AT_1_-R activation leads to calcium signal and the activation of many calcium-dependent kinases in VSMCs, we investigated the effect of a calcium chelator, BAPTA-AM, and specific inhibitors of CaMKII, PKC, and Pyk2. The results demonstrated that the calcium signal and CaMKII play an essential role in all the investigated DUSP expression changes (Supplementary Figure S2D–F), whereas PKC and Pyk2 calcium-dependent kinases play no significant role in the AngII-induced DUSP5 and DUSP6 mRNA-level changes (Supplementary Figure S3). The DUSP10 level seems to be regulated only by PKC, not by Pyk2 activity (Supplementary Figure S3).

EGFR is a receptor tyrosine kinase, which is a key regulator in cardiovascular functions and plays many roles in VSMCs [48]. The stimulation of EGFR leads not just to the activation of Src family kinases and MAPKs promoting VSMC proliferation but also the calcium signal potentiating myogenic tone [49,50]. EGF stimulation was able to induce the DUSP6 mRNA level to a similar extent as the AngII but the EGF was less effective in the regulation of DUSP5 and DUSP10 levels (Figure 2). EGF-induced upregulation was wiped out by small-molecule EGFR kinase inhibitors (AG1478 and gefitinib), whereas the AngII-induced changes were significantly diminished by AG1478 or gefitinib pretreatment (Figure 4A–C). The results regarding the inhibition of matrix metalloproteinases revealed that EGFR transactivation has no exclusive role in the regulation of AngII-induced DUSP mRNA levels, which finding was further confirmed in EGFR gene-silencing experiments ( Figure 5D–F and Figure 7). Our experiments also proved that PDGFR or IGFIR transactivation has no significant role in the AngII-induced DUSP mRNA level changes, and only sorafenib caused a significant reduction in AngII-induced gene expression changes in the case of *DUSP10* (Supplementary Figure S4). The AngII-induced EGFR transactivation depends on calcium signal, calcium-dependent Pyk2 and cytoplasmic Src-like tyrosine kinases [37,51,52]. Among the investigated DUSP isoforms, the *DUSP5* upregulation is, at least partly, EGFR transactivation-dependent since either the inhibition of MMP enzymes by MMP-2/MMP-9 Inhibitor II or the silencing of EGFR by shRNA significantly reduced the effect of AngII stimulation. It is important to note that simultaneous AngII and EGF stimuli cause augmented DUSP levels compared to single AngII treatments (Figure 8), which indicates a synergistic role of EGFR- and AT_1_-R-induced signaling pathways and/or synergism of transcriptional factors in the regulation of DUSP levels. We may consider at least two mechanisms that lead to the upregulation of *DUSP* genes, an EGFR-dependent and an EGFR-independent mechanism. The EGFR transactivation-independent mechanisms are supported by the results that demonstrate less-efficient DUSP5 and DUSP10 upregulation by direct EGF stimulation compared to the AngII effect (Figure 3), and the partial effects of MMP2/9 inhibition and EGFR silencing (Figure 5A–C and Figure 7).

Mitogen-activated protein kinases (MAPKs) are important regulators of numerous cell functions including proliferation, apoptosis, and differentiation. Initially, the DUSP enzymes are considered as negative regulators of MAPK pathways [18], but it is possible that the various DUSPs and changes in their expression level can regulate/orchestrate the pattern of MAPK activation in response to repeated AngII stimuli. The gene expression induction of the various DUSPs in response to AngII stimulation is ultimately mediated by MAPK activity, primarily by ERK1/2 and p38 MAPKs (Supplementary Figure S5). It is possible that the AngII-induced DUSP5, DUSP6 and DUSP10 increase regulates the MAPK signaling in the long term in the VSMCs. The induced DUSPs may alter the MAPK signaling outcome (magnitude and duration) of subsequent stimulations. Both nuclear-localized DUSP5 and cytoplasmic DUSP6 are known to be induced by ERK1/2 activity and they play an important role in the negative feedback loop to limit ERK1/2 activation. DUSP5 specifically interacts with and inactivates ERK1/2 but the DUSP5 itself is also regulated by ERK1/2 activity, i.e., the transcription of DUSP5 mRNA is dependent on the phosphorylation of Elk-1 by ERK1/2 [53]. The overexpression of DUSP5 in cardiomyocytes results in ERK1/2 inactivation and the reduction of agonist-dependent hypertrophy [54], but it was recently demonstrated that although DUSP5 terminates nuclear ERK signaling and anchors ERK in the nucleus, DUSP5 increases the ERK activation in MEF cells [55]. DUSP6 is primarily located in the cytoplasm, binds to the activated ERK1/2, and causes the cytoplasmic retention of the inactivated ERK1/2 in the cytoplasm to play a role in the spatiotemporal regulation of MAPK activity [56]. It was demonstrated that *DUSP6* gene expression and *DUSP6* mRNA stability are controlled by the MEK-ERK1/2 pathways [57]. The DUSP6 KO mice showed increased basal ERK1/2 phosphorylation in multiple tissues and showed increased heart weight; however, this did not result in increased or prolonged ERK1/2 activation in response to stimulation, suggesting that DUSP6 is responsible for the fine-tuning of basal ERK1/2 activity [58]. Based on these findings, it is highly possible that DUSP5 and DUSP6 regulate the nucleo-cytoplasmic shuttling of ERK1/2, together sequestering the dephosphorylated ERK either in the nucleus or in the cytoplasm [18]. Our demonstrated results suggest that these DUSP isoforms can play an essential role in the regulation of MAPK signaling patterns in VSMCs. DUSP10, also called MKP-5, can be found in both the nucleus and cytoplasm, and is capable of regulating multiple MAPK pathways, including JNK, p38, and ERK1/2 MAPKs [59]. Among those pathways, the activated JNK and p38 MAPKs are more effectively dephosphorylated by DUSP10 than the ERK1/2 [60]. Interestingly, DUSP10 not only inactivates ERK MAPKs but also interacts with them. DUSP10 is capable of retaining ERK MAPKs in the cytoplasm and downregulating ERK-dependent transcription [61]. The multiple roles of DUSP10 in the regulation of MAPK signaling are also indicated in a study where the knockdown of DUSP10 inhibited acute EGF-stimulated ERK activation that could be reversed by the pharmacological inhibition of p38 MAPK, suggesting that DUSP10 may modulate crosstalk between the ERK1/2 and p38 MAPK pathways [62]. It is important to note that the expression of DUSP10 is elevated in various diseases, such as atherosclerosis [63], which may be the case in other pathophysiological conditions induced by the over-activation of AT_1_-R. It is also possible that due to the not-exclusive substrate preference of certain DUSPs (i.e., DUSP10) and the crosstalk mechanisms in parallel MAPK pathways, the AngII stimulation-induced DUSPs can suppress or modify certain types of MAPKs, thus shaping the pattern/interplay of the MAPK network after repeated hormone stimuli. The confirmation of this possibility requires further study. In addition, the upregulated DUSP enzymes may regulate not just MAPKs but other proteins, either by dephosphorylating them and/or by binding to them. AngII-activated MAPK signaling also has implications in the phenotypic switching of VSMCs [64]. Since VSMC plasticity is an important factor in the physiological and pathological processes of the vasculature, we find that it is worthwhile to study the role of DUSPs in this context.

Our studies using the pharmacological inhibition of various signaling elements and the gene silencing of EGFR revealed that there is not an exclusive, or predominant, signal transduction pathway in primary rat VSMCs that leads to or may explain the investigated DUSP gene expression changes, and our experimental data shed light on the complex interplay/regulation among the signaling pathways. Our data showed that AngII-induced gene expression regulation is much more complex than we originally thought, due to the multiple signaling pathways that mediate them. The regulation of expression changes is very complex and is probably determined by the interplay of the involved signaling cascades. According to our data, different mechanisms can lead to the expression changes; classical G_q/11_ activation initiated the Ca^2+^-dependent mechanism, which induces EGFR transactivation-dependent and independent mechanisms. Further studies are needed to establish how the contributory signaling pathways could be successfully targeted in the treatment of diseases caused by AT_1_-R over-activation.

The pleiotropic effects of AngII on vascular smooth muscle cells contribute to the development of numerous cardiovascular diseases, such as hypertension, cardiac hypertrophy, and atherosclerosis. Our data provided new insights into the physiology of VSMCs in response to AngII stimulation, and a better understanding of the mechanisms of AT_1_-R-mediated gene expression changes in primary VSMCs can lead to the development of novel types of drugs for the treatment of cardiovascular and other diseases. In addition, the AT_1_-R is a prototypical GPCR, with its pleiotropic action of mechanisms, so the described and revealed mechanisms can be considered valid in the case of other GPCRs in VSMCs.

## Data Availability

Data from Affymetrix GeneChip experiments will be available after publication.

## Supplementary Materials

The following are available online at https://www.mdpi.com/article/10.3390/cells10123538/s1, Figure S1: Verification of the basic properties of the isolated VSMCs; Figure S2. Role of calcium signal in gene expression changes of DUSP isoforms in VSMCs; Figure S3. Evaluation of the contribution of PKC signal transduction in the AngII- and EGF-induced gene expression changes of DUSP isoform in VSMCs; Figure S4. Effect of PDGFR and VEGFR and IGF-1R tyrosine kinase inhibitors on AngII and EGF mediated induction of DUSP expression in vascular smooth muscle cells; Figure S5. Importance of MAPK cascade activation in AngII mediated upregulation of DUSP isoforms in vascular smooth muscle cells.
