# Supplementary material for: Characterization of Type 1 Angiotensin II Receptor Activation Induced Dual-Specificity MAPK Phosphatase Gene Expression Changes in Rat Vascular Smooth Muscle Cells
Source: Cells. 2021 Dec 15;10(12):3538. doi: 10.3390/cells10123538 (PMC8700539; doi:10.3390/cells10123538)
Supplement: Supplementary file 1 [file cells-10-03538-s001.zip › cells-1484224-supplementary.pdf]

Supplementary Materials: The following are available online at www.mdpi.com/xxx/s1,

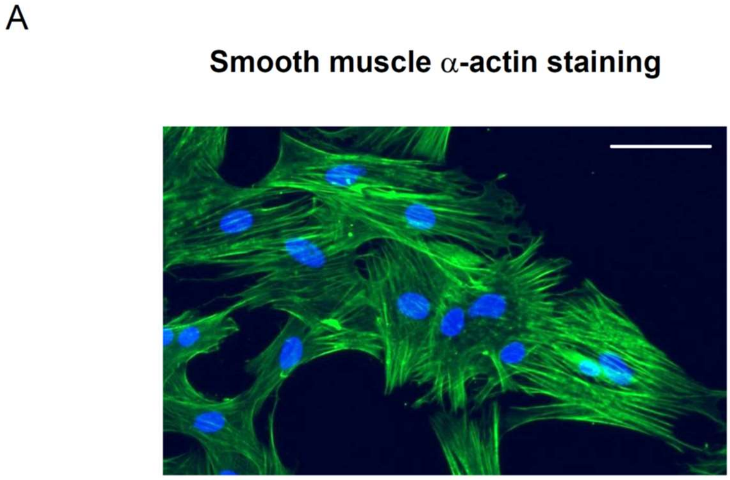

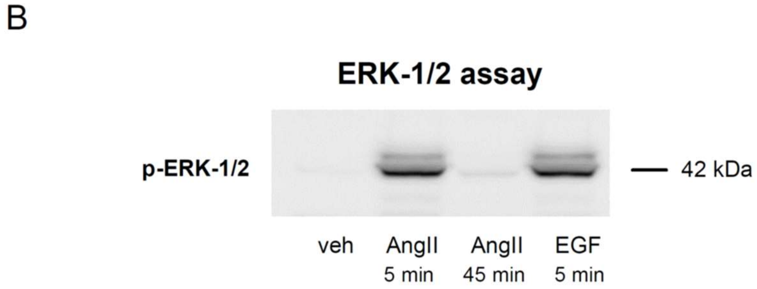

Supplementary Figure S1. Verification of the basic properties of the isolated VSMCs. (A) Immunostaining of rat primary VSMC culture with smooth muscle α-actin (green), the nuclei were stained by DAPI (blue). Bar: 50 μm. (B) ERK-1/2 MAPK activation. Rat aortic VSMCs were exposed to vehicle, 100 nM AngII or 3 μM TRV120023 (TRV) for 5 min. The western blot is a representative of 3 independent experiments.

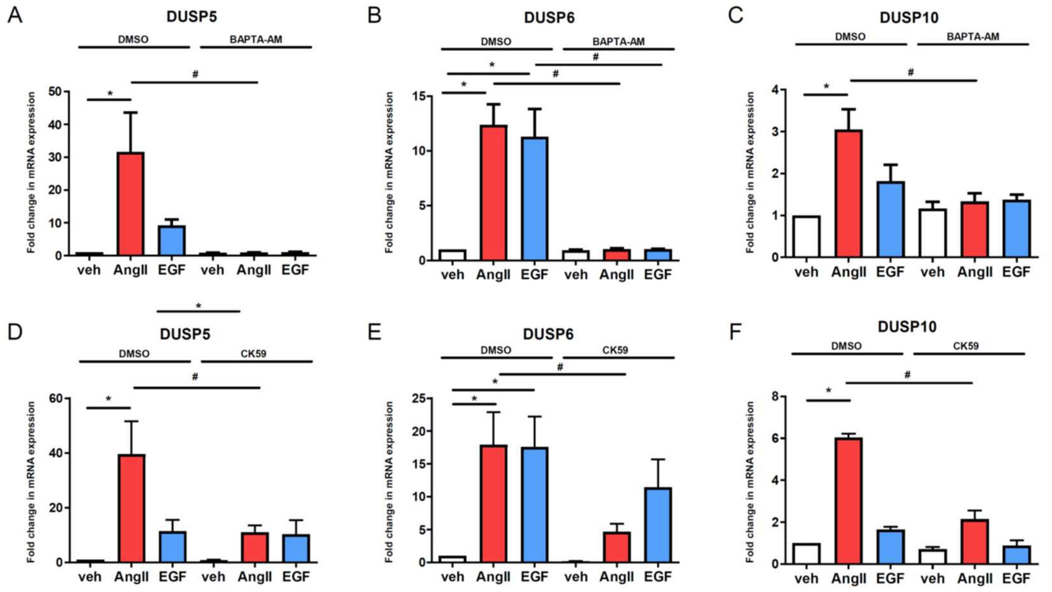

Supplementary Figure S2. Role of calcium signal in gene expression changes of DUSP isoforms in VSMCs. Serum depleted VSMCs were incubated with 50 μM BAPTA-AM or DMSO as control for 10 minutes (A-C) or pretreated with 50 μM CamKII inhibitor, CK59 beside a DMSO treated control group for 30 minutes (D-F), then the cells were exposed to either 100 nM AngII (red columns) or 50 ng/ml EGF (blue columns) or vehicle (white columns) for 2 hours. RNA was isolated from VSMCs, then converted to cDNA. cDNA levels of DUSP5 (A and D), DUSP6 (B and E) and DUSP10 (C and F) were measured by qRT-PCR. Standardization was made against the *GAPDH* housekeeping gene. The mRNA levels were normalized to values of DMSO vehicle samples and expressed as fold change. Mean values ± S.E. are shown (n = *3-6*). Significance was determined with multiple linear regression. $p < 0.05$ was considered as statistically significant. *: Statistically significant from vehicle stimulation. #: Statistically significant from DMSO pretreated agonist induced response. The values are from three to six independent experiments.

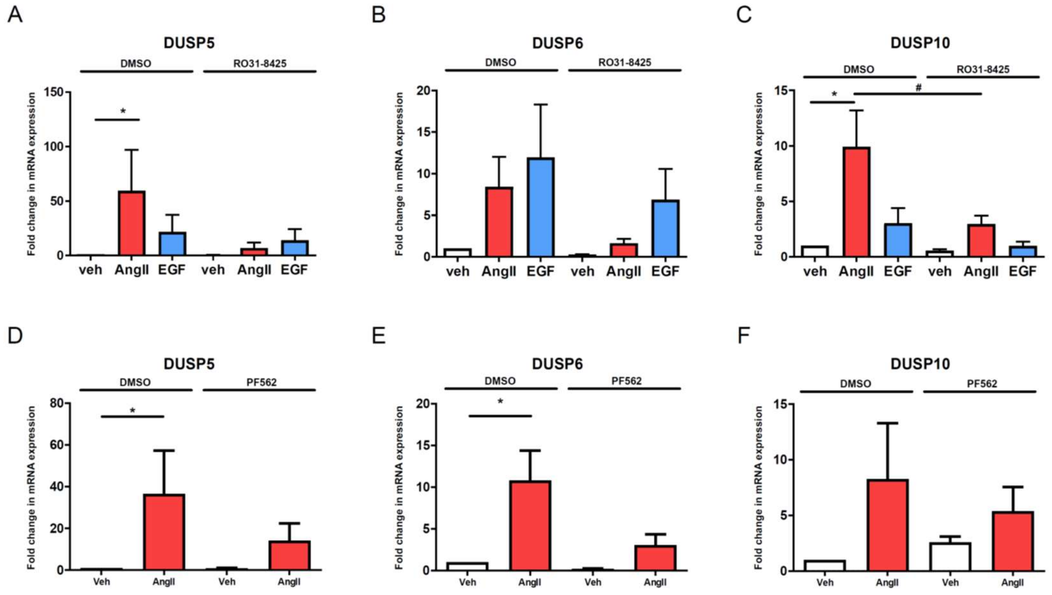

Supplementary Figure S3. Evaluation of the contribution of PKC signal transduction in the AngII- and EGF-induced gene expression changes of DUSP isoform in VSMCs. Cells were serum starved and then pretreated with 1 μM of PKC inhibitor RO31-8425 or DMSO as control for 10 minutes (A-C),

others were incubated with 1 μM PF-562271, Pyk2 inhibitor beside DMSO treated control groups (D-F), then they were exposed to either 100 nM AngII (red columns) or 50 ng/ml EGF (blue columns, A-C) or vehicle (white columns) for 2 hours. RNA was isolated from VSMCs, then converted to cDNA. cDNA levels of DUSP5 (A and D), DUSP6 (B and E) and DUSP10 (C and F) were measured by qRT-PCR. Standardization was made against the *GAPDH* housekeeping gene. The mRNA levels were normalized to values of DMSO vehicle samples and expressed as fold change. Mean values ± S.E. are shown (*n* = *3*). Significance was determined with multiple linear regression. $p < 0.05$ was considered as statistically significant. *: Statistically significant from vehicle stimulation. #: Statistically significant from DMSO pretreated agonist induced response. The values are from three independent experiments.

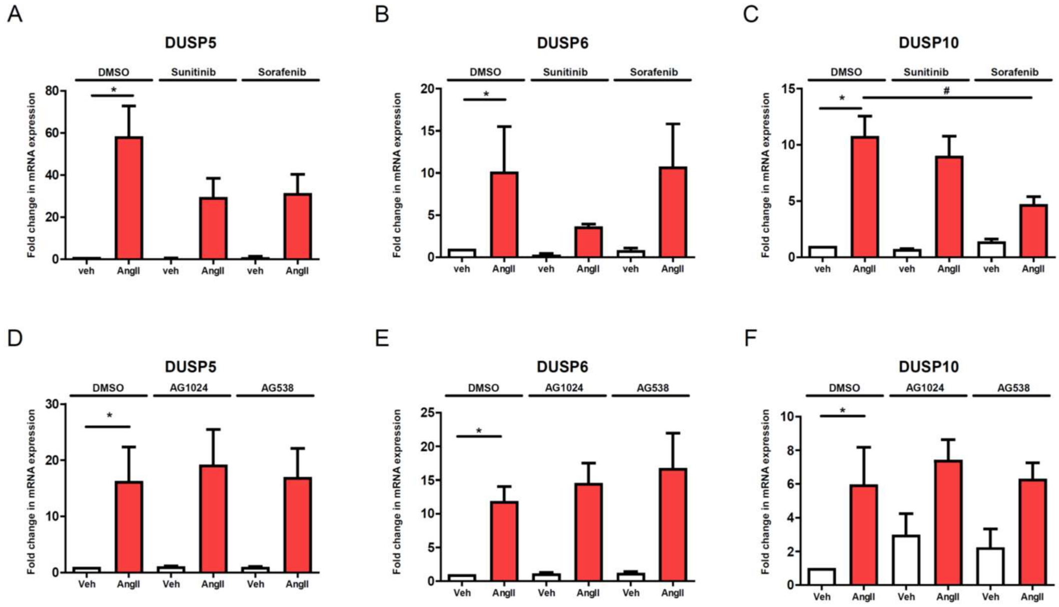

Supplementary Figure S4. Effect of PDGFR and VEGFR and IGF-1R tyrosine kinase inhibitors on AngII and EGF mediated induction of DUSP expression in vascular smooth muscle cells. Serum deprived VSMCs were treated with either DMSO or 5 μM Sunitinib or 5 μM Sorafenib (A-C) or 1-1 μM of IGF-1R inhibitors AG1024 and AG538 (D-F) prior to stimulation with either vehicle (white columns) or 100 nM AngII (red columns) or 50 ng/ml EGF (blue columns) for 2 hours. mRNA levels of DUSP5 (A and D), DUSP6 (B and E) and DUSP10 (C and F) were measured by qRT-PCR. Standardization was made against the *GAPDH* housekeeping gene. The mRNA levels were normalized to values of DMSO vehicle samples and expressed as fold change. Mean values ± S.E. are shown (*n* = *3-4*). Significance was determined with multiple linear regression. $p < 0.05$ was considered as statistically significant. *: Statistically significant from vehicle stimulation. #: Statistically significant from DMSO pretreated agonist induced response. The values are from three to four independent experiments.

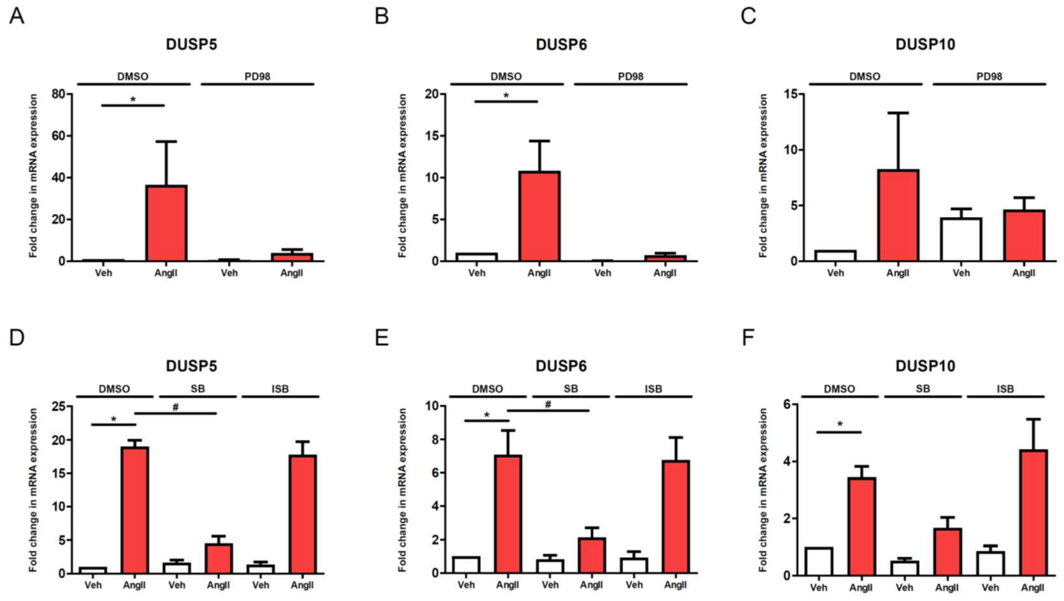

Supplementary Figure S5. Importance of MAPK cascade activation in AngII mediated upregulation of DUSP isoforms in vascular smooth muscle cells. Serum starved VSMCs were pretreated with 20 μM PD98059 (PD98) MEK inhibitor (A-C) or 50-50 μM of p38 MAPK inhibitor SB202190 (SB) or its inactive analog SB202474 (ISB) (D-F) beside DMSO treated control groups for 30 minutes, then stimulated with either vehicle (white columns) or 100 nM AngII (red columns) for 2 hours. mRNA levels of DUSP5 (A and D), DUSP6 (B and E) and DUSP10 (C and F) were measured by qRT-PCR. Standardization was made against the *GAPDH* housekeeping gene. The mRNA levels were normalized to values of DMSO vehicle samples and expressed as fold change. Mean values ± S.E. are shown (n = 3). Significance was determined with multiple linear regression. $p < 0.05$ was considered as statistically significant. *: Statistically significant from vehicle stimulation. #: Statistically significant from DMSO pretreated agonist induced response. The values are from three independent experiments.
